# Robust 3D point cloud registration based on bidirectional Maximum Correntropy Criterion

**DOI:** 10.1371/journal.pone.0197542

**Published:** 2018-05-25

**Authors:** Xuetao Zhang, Libo Jian, Meifeng Xu

**Affiliations:** 1 The School of Electronic and Information Engineering, Xi’an Jiaotong University, Xi’an 710049, China; 2 The Second Affiliated Hospital of Xi’an Jiaotong University, Xi’an 710004, China; Huazhong University of Science and Technology, CHINA

## Abstract

This paper presents a robust 3D point cloud registration algorithm based on bidirectional Maximum Correntropy Criterion (MCC). Comparing with traditional registration algorithm based on the mean square error (MSE), using the MCC is superior in dealing with complex registration problem with non-Gaussian noise and large outliers. Since the MCC is considered as a probability measure which weights the corresponding points for registration, the noisy points are penalized. Moreover, we propose to use bidirectional measures which can maximum the overlapping parts and avoid the registration result being trapped into a local minimum. Both of these strategies can better apply the information theory method to the point cloud registration problem, making the algorithm more robust. In the process of implementation, we integrate the fixed-point optimization technique based on the iterative closest point algorithm, resulting in the correspondence and transformation parameters that are solved iteratively. The comparison experiments under noisy conditions with related algorithms have demonstrated good performance of the proposed algorithm.

## 1 Introduction

The development of scanning equipment makes the acquisition of 3D point cloud possible [1, 2]. Point cloud registration problem has attracted considerable attentions in computer vision [3, 4]. The goal of registration is to find the correspondence and estimate the optimal rigid transformation between two point clouds. The conventional methods based on the iterative closest point (ICP) [5, 6] algorithm have been widely used for the advantages of high speed and accuracy. Usually, such methods build a global hard correspondence which are efficient but not robust enough with the presence of various noise, outliers and partial difference.

To overcome the above disadvantage, two kinds of improvements have been applied by researchers. One is to replace the hard assignment with soft assignment, which uses one-to-more correspondences instead of one-to-one mapping [7, 8]. For example, Liu et al. [9] added the expectation maximization principle to the ICP, and used the SoftAssign for registration of overlapping point sets. Jian et al. [10] modeled point sets as probability density functions (PDF) by Gaussian mixture model. The distance between two global distributions is minimized for fitting two point sets with some smooth transformation constraints. Similar idea was used by Zhou et al. [11] for non-rigid point set registration. These methods are accurate and robust. But they need to estimate the correspondence from each point to all points. The computational complexity is very high. The other way is to select part of reliable point-pairs to estimate the transformation. The straightforward solution is to add a penalty term for the corresponding points with greater distance [12]. In order to balance the penalty intensity automatically, a parameter related with the overlapping ratio was applied into a series of methods [13–15] to adjust the trimming and matching ratio. These methods are effective to overcome the challenge of partial difference. But in reality, when varied kinds of non-ideal conditions occur together, they are hard to deal with them jointly.

Most traditional registration algorithms build the distance measure based on the mean square error (MSE). In information theoretic learning (ITL) [16, 17], researchers have noticed the poor performance of MSE to process non-Gaussian data. Instead, correntropy, a non-second statistical measure, has shown significant potential in the general robust learning and signal processing [18–21]. The core reason for its robustness is that the correntropy directly estimates the probability of how similar the two random variables are. The adverse effects of noise and outliers are automatically eliminated by assigning small weights. The related work is a shape matching method [22] based on the Maximum Correntropy Criterion(MCC), which is equivalent to minimizing C-Loss between two point sets. This work has shown some superior in dealing with non-Gaussian noise and large outliers. But the algorithm assumes the correspondences between two point sets should be assigned in advance. Moreover the result’s stability should be improved. More recently, Xu et al. [23] employed the correntropy and Yang et al. [24] employ a non-second statistical measure in kernel space under the ICP framework, which can establish the correspondence and do the matching concurrently. However their algorithms are easily converge to partial noisy points. Later, we will present some registration results in [23] in the experiments for comparison.

Different from the above methods, this paper presents a robust point cloud registration algorithm based on the bidirectional Maximum Correntropy Criterion (BiMCC). Since the MCC is considered as a probability measure which weights the correspondence of point sets for registration, the noisy points are penalized. Moreover, using bidirectional measure can maximum the overlapping parts and avoid the registration result being trapped into a local minimum. Both of these two strategies can better apply the information theory method to the registration problem, making the algorithm more robust. In the process of algorithm implementation, we integrate the fixed-point optimization technique into the ICP framework, resulting in the correspondence and transformation parameters that are solved jointly. The comparison experiments under noisy conditions with related algorithms have demonstrated good performance of the proposed algorithm.

The rest of the paper is structured as follows. In Section 2, we give a brief review of correntropy. Following that, Section 3 proposes a novel formulation and optimization algorithm for noisy point cloud registration based on the BiMCC. In Section 4, we present experimental results tested on the benchmark data set to demonstrate the performance. Section 5 concludes this paper.

## 2 Brief review of correntropy

In real world, signals are generally captured from unconstrained scenarios by sensors, which brings big challenges for computer processing. The point cloud captured from 3D space has unpredictable nature errors. Some errors may be with large magnitude as outliers, some of them may be small but with arbitrary distributions. Therefore, a robust cost function should have the ability to filter out all kinds of errors. Today, the MSE is the most widely used in cost function for registration. However, as a standard second order statistics, it is a good solution only for processing Gaussian noise. Recently, correntropy is proposed in ITL to handle non-Gaussian noises and large outliers, which has widely and successfully applications in signal processing and machine learning. Essentially, correntropy is a second order statistical measure in kernel space, which corresponds to a non-second order measure in original space.

Given two arbitrary random variables *X* and *Y*, the correntropy is defined as

$$\begin{array}{c} V_{\sigma }\left(X,Y\right)=E\left[\kappa_{\sigma }\left(X-Y\right)\right], \end{array}$$ (1)

where *κ*(⋅) stands for a kernel function, satisfying *κ*(*x*) ≥ 0 and $\int_{-\infty }^{\infty }\kappa \left(x\right)dx=1$. *E*[⋅] denotes the mathematical expectation. In this work, without mentioned otherwise, the kernel function is selected as a Gaussian kernel, given by

$$\begin{array}{c} \kappa_{\sigma }\left(x\right)=\exp\left(-x^{2}/2\sigma^{2}\right), \end{array}$$ (2)

where *σ* denotes the kernel bandwidth.

In a practical situation, the joint probability density function is usually unknown, and one has to estimate it from a finite number of data pair ${\left(x_{i},y_{i}\right)}_{i=1}^{N}$. Based on the Parzen window approach, the sample estimator of correntropy is simply estimated as

$$\begin{array}{c} {\hat{V}}_{\sigma }\left(X,Y\right)=\frac{1}{N}\sum_{i=1}^{N}\kappa_{\sigma }\left(x_{i}-y_{i}\right), \end{array}$$ (3)

We can see from the Eq (3) that correntropy is directly related to the probability of how similar two random variables along the line *X* = *Y* and in a neighborhood of the joint space controlled by the kernel bandwidth. This is the reason that correntropy weights the correspondence of points.

## 3 Proposed algorithm

In this section, we first define the bidirectional correntropy for two point cloud data, and propose the rigid registration formulation for noisy point cloud based on the bidirectional Maximum Correntropy Criterion (BiMCC). And then we develop the optimization method to estimate optimal parameters. Finally, the theory analysis is presented with the algorithm property.

### 3.1 Problem formulation

Given two point clouds in 3D space, denoted as a model point cloud $X≜{\left\{{\vec{x}}_{i}\right\}}_{i=1}^{N_{x}}\left({\vec{x}}_{i}\in R^{3\times 1}\right)$ and a target point cloud $Y≜{\left\{{\vec{y}}_{j}\right\}}_{j=1}^{N_{y}}\left({\vec{y}}_{j}\in R^{3\times 1}\right)$, the goal for registration is to build up the correspondence and calculate the rigid transformation to achieve the best alignment with two point clouds. We assume the corresponding point for ${\vec{x}}_{i}$ in the opposite point cloud is ${\vec{y}}_{c(i)}$, using *c*(⋅) to denote the corresponding index. The registration process is to rigid transform the point ${\vec{x}}_{i}$ by a rotation matrix **R** and translation vector $\vec{t}$ to ${\vec{y}}_{c(i)}$, then the error between two matched points is

$$\begin{array}{c} e_{x_{i}}=R{\vec{x}}_{i}+\vec{t}-{\vec{y}}_{c(i)}, \end{array}$$ (4)

For each point in *X*, it can be found one corresponding point in *Y* by the nearest neighbor searching. These corresponding points compose a new data set denoted as $\tilde{Y}$, which has the same number and order with *X*. If model point set and its corresponding point set are considered as two random variables with noise and outliers, we measure their correntropy as:

$$\begin{array}{ccc} {\hat{V}}_{\sigma }\left(X,\tilde{Y},R,\vec{t}\right) & = & \frac{1}{N_{x}}\sum_{i=1}^{N_{x}}\kappa_{\sigma }\left(e_{x_{i}}\right)\\ & = & \frac{1}{N_{x}}\sum_{i=1}^{N_{x}}\exp\left(\frac{-{∥R{\vec{x}}_{i}+\vec{t}-{\vec{y}}_{c(i)}∥}^{2}}{2\sigma^{2}}\right). \end{array}$$ (5)

It is easy to know that $\tilde{Y}\subset Y$. So, when we only consider one corresponding direction, part of the points in *Y* will be ignored in the formulation. Without a good initial position, most of the *Y* are wrong at the beginning. Especially with the presence of noise, the distance between model points and noisy points may be smaller compared with other true correspondences. In that case, these noisy points are trusted for registration which causes the result being stick to partial noisy region.

To avoid this situation, we define the bidirectional correntropy for two point clouds. Correspondingly, for each point in *Y*, it can be found one corresponding point in *X* by the nearest neighbor searching. These corresponding points compose a new data set denoted as $\tilde{X}$, which has the same number and order with *Y*. It can be understood as we construct two big point sets $\left\{X,\tilde{X}\right\}$ and $\left\{\tilde{Y},Y\right\}$, respectively, with the same point number *N* = *N*_*x*_ + *N*_*y*_. With the help of more correspondence between $\left\{X,\tilde{X}\right\}$ and $\left\{\tilde{Y},Y\right\}$, the weight of local registration will be reduced. The result leans to a global registration.

Then, the bidirectional correntropy is formulated as:

$$\begin{array}{ccc} & & {\hat{V}}_{\sigma }\left(\left\{X,\tilde{X}\right\},\left\{Y,\tilde{Y}\right\},R,\vec{t}\right)\\ & & \;=\frac{1}{N}\left(\sum_{i=1}^{N_{x}}\exp\left(\frac{-{∥R{\vec{x}}_{i}+\vec{t}-{\vec{y}}_{c(i)}∥}^{2}}{2\sigma^{2}}\right)+\sum_{j=1}^{N_{y}}\exp\left(\frac{-{∥R{\vec{x}}_{d(j)}+\vec{t}-{\vec{y}}_{j}∥}^{2}}{2\sigma^{2}}\right)\right). \end{array}$$ (6)

The two terms in Eq (6) are the sum of the forward distance and backward distance between two point sets respectively. For each term, it is formulated as a non-second order manner. This formulation also denotes a distance measure between two point sets. To minimum the distance measure is equal to maximum the bidirectional correntropy, call as BiMCC. Thus, the proposed the cost function for the point cloud registration based on BiMCC is

$$\begin{array}{ccc} & & \text{max}{\hat{V}}_{\sigma }\left(\left\{X,\tilde{X}\right\},\left\{Y,\tilde{Y}\right\},R,\vec{t}\right)\\ & & s.t.\;R^{T}R=I_{3},\;\det\left(R\right)=1. \end{array}$$ (7)

Note that since **R** is a rotation matrix, it should satisfy the constrained conditions **R**^*T*^ **R** = **I**_3_ and det(**R**) = 1. The constraint about rotation matrix **R** should be added, when we maximize the cost function.

### 3.2 Parameter estimation

In the above cost function Eq (7), there are two kinds of parameters to be optimized: 1) the reconstructed point sets $\tilde{X}$ and $\tilde{Y}$. It is equal to find the one-to-one corresponding index *c*(*i*) and *d*(*j*) between two point sets, 2) the rigid transformation parameters **R** and $\vec{t}$. Actually, these two kinds of parameters are close related with each other. When one of them has been known, the other is easily to be obtained. Moreover, the parameter estimation can be solved like the ICP algorithm under an iteration framework. We call the proposed algorithm as ICP-BiMCC, which is presented as follows:

**Step 1**: Given the (*k* − 1)^*th*^ transformation parameters $\left(R_{k-1},{\vec{t}}_{k-1}\right)$, the bidirectional corresponding points between two point-sets are found by the nearest neighbor searching:

$$\begin{array}{ccc} & & c_{k}\left(i\right)=\arg\min_{j\in 1,2,...,N_{y}}\left(R_{k-1}{\vec{x}}_{i}+{\vec{t}}_{k-1}-{\vec{y}}_{j}\right),\;\;i=1,...,N_{x}\\ & & d_{k}\left(j\right)=\arg\min_{i\in 1,2,...,N_{x}}\left(R_{k-1}{\vec{x}}_{i}+{\vec{t}}_{k-1}-{\vec{y}}_{j}\right),\;\;j=1,...,N_{y}. \end{array}$$ (8)

The above **Step 1** finds the closet point in another point set for each point. Some conventional searching strategies like Delaunay triangulation and k-d tree are able to be used in advance to speed up the computation.

**Step 2**: According to the current correspondence $\left\{i,d_{k}{\left(j\right)}_{j=1}^{N_{y}}\right\}$ and $\left\{c_{k}{\left(i\right)}_{i=1}^{N_{x}},j\right\}$, two point sets $U={\left\{{\vec{u}}_{i}\right\}}_{i=1}^{N}$ and $V={\left\{{\vec{v}}_{i}\right\}}_{i=1}^{N}$ are redefined as

$$\begin{array}{c} {\vec{u}}_{i}=\left\{ \begin{array}{cc} {\vec{x}}_{i}, & 1\leq i\leq N_{x},\\ {\vec{x}}_{d_{k}\left(i\right)}, & N_{x}+1\leq i\leq N. \end{array}\right. \end{array}$$

$$\begin{array}{c} {\vec{v}}_{i}=\left\{ \begin{array}{cc} {\vec{y}}_{c_{k}\left(i\right)}, & 1\leq i\leq N_{x},\\ {\vec{y}}_{i}, & N_{x}+1\leq i\leq N. \end{array}\right. \end{array}$$

Then the transformation parameters are computed by maximizing the following function:

$$\begin{array}{c} F\left(R,\vec{t}\right)=\frac{1}{N}\sum_{i=1}^{N}\exp\left(\frac{-{∥R{\vec{u}}_{i}+\vec{t}-{\vec{v}}_{i}∥}^{2}}{2\sigma^{2}}\right). \end{array}$$ (9)

Firstly, taking the derivative of Eq (9) with respect to $\vec{t}$, we have:

$$\begin{array}{ccc} & & \frac{\partial F(R,\vec{t})}{\partial \vec{t}}=0\\ & & ⟹\frac{1}{N\sigma^{2}}\sum_{i=1}^{N}\exp\left(\frac{-{∥R{\vec{u}}_{i}+{\vec{t}}^{*}-{\vec{v}}_{j}∥}^{2}}{2\sigma^{2}}\right)\left(R{\vec{u}}_{i}+\vec{t}-{\vec{v}}_{i}\right)=0\\ & & ⟹{\vec{t}}^{*}=\frac{\sum_{i=1}^{N}\kappa_{\sigma }\left(e_{i}\right)\left({\vec{v}}_{i}-R{\vec{u}}_{i}\right)}{\sum_{i=1}^{N}\kappa_{\sigma }\left(e_{i}\right)}, \end{array}$$ (10)

where $e_{i}=R{\vec{u}}_{i}+\vec{t}-{\vec{v}}_{i}$.

To simplify the cost function, we substitute ${\vec{t}}^{*}$ into Eq (10) and let

$$\begin{array}{c} {\vec{p}}_{i}≜{\vec{u}}_{i}-\sum_{i=1}^{N}\kappa_{\sigma }\left(e_{i}\right){\vec{u}}_{i}/\sum_{i=1}^{N}\kappa_{\sigma }\left(e_{i}\right),\;{\vec{q}}_{i}≜{\vec{v}}_{i}-\sum_{i=1}^{N}\kappa_{\sigma }\left(e_{i}\right){\vec{u}}_{i}/\sum_{i=1}^{N}\kappa_{\sigma }\left(e_{i}\right). \end{array}$$ (11)

Intuitively, point sets *P* and *Q* are normalized by weighted centroid.

Therefore,

$$\begin{array}{c} R^{*}=\text{arg}\underset{R^{T}R=I_{3},\det\left(R\right)=1}{\text{max}}\frac{1}{N}\sum_{i=1}^{N}\exp\left(\frac{-{∥R{\vec{p}}_{i}-{\vec{q}}_{i}∥}^{2}}{2\sigma^{2}}\right). \end{array}$$ (12)

It is difficult to solve the constraint optimization problem by simple technique. But, Arun et al. [25] has presented in the ICP algorithm to solve for the rotation matrix with respect to the MSE, by calculating the matrix **H** and its singular value decomposition (SVD). The difference is that our cost function is based on the MCC, the modified matrix **H** is

$$\begin{array}{c} H=\sum_{i=1}^{N}{\vec{p}}_{i}\kappa_{\sigma }\left(e_{i}\right){\vec{q}}_{i}^{T}. \end{array}$$ (13)

And we make the SVD of **H**, it becomes: **H** = **U**Λ**V**. Then we get the optimal R*:

$$\begin{array}{c} R^{*}=VDU^{T}, \end{array}$$ (14)

where,

$$\begin{array}{c} D=\{\begin{array}{cc} I_{3}, & \det(H)>0,\\ diag(1,1,-1), & \det(H)<0. \end{array} \end{array}$$

Finally, with the known parameters R*, we can calculate the translation parameter ${\vec{t}}^{*}$ according to Eq (10). R* and ${\vec{t}}^{*}$ are the current transformation under the last *k* − 1 time result. Thus, the transformation on the original template should be updated. Update **R**_*k*_ and ${\vec{t}}_{k}$ by:

$$\begin{array}{c} R_{k}=R^{*}R_{k-1},\;{\vec{t}}_{k}=R^{*}{\vec{t}}_{k-1}+{\vec{t}}^{*} \end{array}$$

The above **Steps 1** and **2** are repeated until satisfying the stop criteria.

We can see that both the optimal parameters R* and ${\vec{t}}^{*}$ depend on the kernel function *κ*_*σ*_(*e*_*i*_). Whereas, *e*_*i*_ is related to the parameters **R** and $\vec{t}$. So it is a fixed-point equation which can be solved by a fixed-point iterative algorithm. For the solution of the algorithm, we assume that the kernel function is fixed as the last iteration $\kappa_{\sigma }\left(e_{i}\right)=\kappa_{\sigma }\left(R_{k-1}{\vec{u}}_{i}+{\vec{t}}_{k-1}-{\vec{v}}_{i}\right)$. Since after a few iterations, there is no much difference of parameters between two continuous iterations, this approximation will not affect the final registration result. So the complete algorithm only has one iterative layer. Algorithm 1 summarizes the process of the proposed ICP-BiMCC algorithm for rigid point cloud registration. There are some effective ways [26] to set optimal initialization, which are not discussed in this paper. Here, we use the identity matrix as the initial parameter.

**Algorithm 1** ICP-BiMCC

**Input:** model point cloud ${\left\{{\vec{x}}_{i}\right\}}_{i=1}^{N_{x}}$ and data ${\left\{{\vec{y}}_{j}\right\}}_{j=1}^{N_{y}}$

**Output:** transformation **R** and $\vec{t}$, correspondences $\left\{i,d{\left(j\right)}_{j=1}^{N_{y}}\right\}$ and $\left\{c{\left(i\right)}_{i=1}^{N_{x}},j\right\}$

1: Initialization: Set **R**_0_ = **I**_3_ and ${\vec{t}}_{0}=0$, and randomly initialized *σ*_0_

2: **for** *k* = 1, 2, …, *K* **do**

3:  Set up the correspondence *c*_*k*_(*i*) and *d*_*k*_(*j*) by nearest neighbor searching;

4:  Compute the kernel *κ*_*σ*_(*e*_*k*_) by **R**_*k*−1_ and ${\vec{t}}_{k-1}$;

5:  Reconstruct the point sets **P** and **Q**;

6:  Solve the rotation matrix R* by Eq (14);

7:  Solve the translation vector ${\vec{t}}^{*}$ by Eq (10);

8:  Update the transformation parameters **R**_*k*_ and ${\vec{t}}_{k}$;

9:  Compute the MSE *e*_*k*_;

10: Set the kernel width *σ*_*k*_;

11: Until ‖*e*_*k*_ − *e*_*k*−1_‖ < *ε*.

 **return**

### 3.3 Theory analysis

Compared with the conventional ICP algorithm, there are two main improvements of the proposed ICP-BiMCC algorithm. We firstly use a bidirectional measure to build the correspondence, and then compute the rigid transformation by a non-second order measure: correntropy. In this section, we analysis the reasons why the ICP-BiMCC algorithm is more robust by these two improvements.

#### 3.3.1 Computing the transformation

In each iteration, when correspondence has been established, the one-to-one point-pairs are selected as candidates for computing the rigid transformation. The ICP algorithm uses all of them equally to estimate transformation parameters. But when there are some noise or outliers, the wrong correspondences will drag the global transformation to a bad position. Instead, the ICP-BiMCC algorithm utilises the correntropy measure. As we mentioned, the correntropy is directly related to the probability of how similar two random variables are. Intuitively, we can see the distribution of Gaussian function *κ*_*σ*_(*e*). When e locates outside of the space around 0 vector, the value reduces quickly. That means only when the two corresponding points are close enough, they could be used reliably for estimating transformation parameters. Otherwise, they have little influence for the result. Since the noisy points and outliers are usually far away from the model points, the proposed algorithm can avoid the bad effects caused by noise and outliers in theory.

Here, the kernel bandwidth *σ* plays an important role in controlling the weight of each corresponding point’s contribution. It affects the range which distinguishes the inliers and noisy points. Since the majority correspondence are not accurate at the beginning, all points should be used without obvious differences. Here we set a big bandwidth. Gradually, after a few iterative steps one-to-one correspondences are built accurately. Noisy points should be filtered out by small bandwidth. Therefore, the kernel bandwidth is varied from large to small. It is corresponding to the coarse-to-fine registration strategy. We employ the Silvermans rule [18] in Eq (15) to adjust the kernel width automatically.

$$\begin{array}{c} \sigma^{2}=1.06\times min\left\{\sigma_{E},\frac{D}{1.354}\right\}\times {\left(n\right)}^{-1/5}, \end{array}$$ (15)

where *σ*_*E*_ is the standard deviation of ${∥e_{i}∥}_{2}^{2}$ and *D* is the interquartile range. We can see that *σ* is adjusted according to the current registration’s error, which conforms to the above analysis. Without mentioned otherwise, we apply this chosen principle.

#### 3.3.2 Building bidirectional correspondence

**Fig 1:**
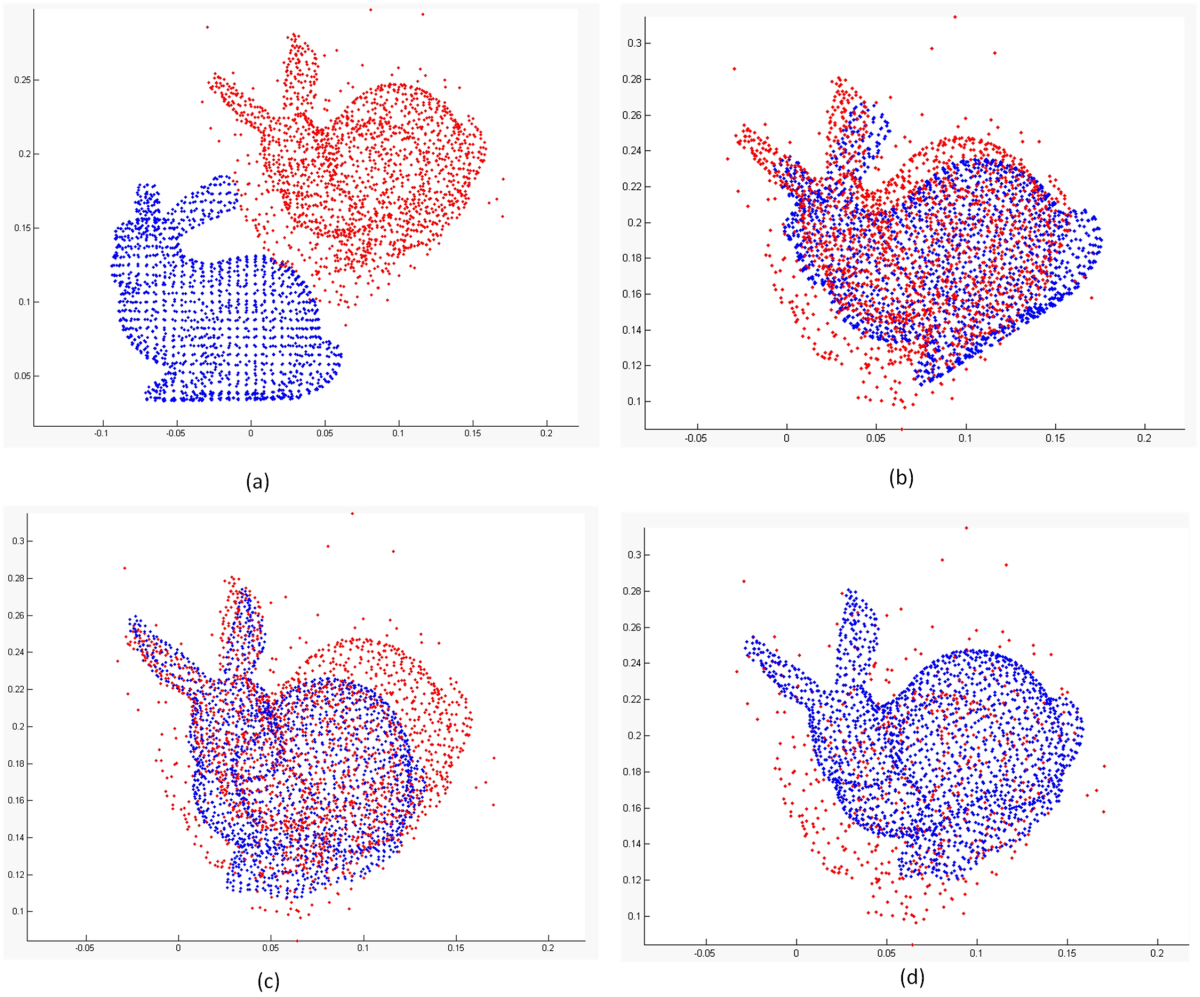
Registration results on noisy ‘bunny’. (a) Initialization. (b) result of ICP. (c) result of ICP-MCC. (d) result of ICP-BiMCC.

In each iteration, the model point cloud is transformed by the estimated parameters to a new position. The correspondences are built according to the nearest neighbor principle in 3D space. Now, we assume that target point cloud is noisy. Some of points are disturbed by a non-zero mean noise but with small variance. It’s can be imagined that these noisy points are similar with the local shape of model point cloud, such as Fig 1. We consider this situation when a certain percentage of model points is matched to noisy data, other points are mismatched. In this case, if we only build a single directional correspondence for every model point, some of them still find the noisy data with very short distance values, and others find the wrong correspondences with big distance values. Based on the above analysis of MCC, the latter will be considered as noisy points which has no contribution for updating the rigid transformation. Thus, the iterative process terminate, and the registration is trapped into a local minimum as Fig 1(c). Whereas, if we use bidirectional measure to build the correspondence, the most points in target point set will find the right correspondences relatively with model points, which weaken the bad influence caused by noise. Therefore, using bidirectional measures between two point sets can maximum the matching overlaps to reach a global optimization as Fig 1(d).

## 4 Experimental results

In this section, we compare the performance among the ICP algorithm [5], ICP-MCC algorithm [23] and our ICP-BiMCC algorithm. The registration precision is reported by the MSE between corresponding points. An alternative way to measure the performance is the errors between estimated values and the ground truth simulation parameters. For rigid transformation, they are defined as $\varepsilon_{R}=\sqrt{{∥R-R_{G}∥}^{2}}$ and $\varepsilon_{\vec{t}}=\sqrt{{∥\vec{t}-{\vec{t}}_{G}∥}^{2}}$. The experiments are conducted on the public and standard datasets [27] and [28–30]. The following experiments are tested on the noisy data and partial overlapping data, respectively.

### 4.1 Testing on noisy data

In this part, the 3D point clouds ‘Bunny’, ‘Dragon’ and ‘Happy Buddha’ data from S1 Dataset in [27] are tested. The original data is used as model point cloud, and the target point cloud is simulated as the following ways. The model data is firstly rotated by 25 degree around three axis and translated by [0.1, 0.1, 0.1]^*T*^. Then 20% points are selected randomly to be added with Gaussian noise *N*(0, 0.02). 10% points are selected randomly to be added with non-zero mean Gaussian noise *N*(0.003, 0.018). Since the noise is randomly added to the testing data, we repeat every experiment condition for 10 times. The statistical quantitative results: MSE, *E*_**R**_ and $E_{\vec{t}}$ of three point clouds are shown in Table 1. Since the MSE can only deal with global Gaussian noise, the performance is poor of the ICP algorithm. And the ICP-MCC algorithm can filter out some noise and outiers. But they are misled by those non-zero mean noise, so it achieves a local registration. It can be seen that all registration errors of our ICP-BiMCC algorithm are the smallest. The ICP-BiMCC algorithm has the highest robustness and accuracy to noisy data.

**Table 1:** The MSE, *E*_*R*_ and $E_{\vec{t}}$ comparison of point clouds.

Point cloud	Error	ICP	ICP-MCC	ICP-BiMCC
‘Bunny’	MSE(×10^−3^)	5.905	6.05	**1.63**
	*ε*_**R**_(×10^−3^)	345.415	285.985	**8.545**
	$\varepsilon_{\vec{t}}\left(\times 10^{-3}\right)$	27.761	30.77	**1.34**
‘Dragon’	MSE(×10^−3^)	4.23	4.29	**0.889**
	*ε*_**R**_(×10^−3^)	61.71	89.286	**3.89e-5**
	$\varepsilon_{\vec{t}}\left(\times 10^{-3}\right)$	25.8	23.15	**7.24e-3**
‘Happy Buddha’	MSE(×10^−3^)	4.14	3.63	**1.18**
	*ε*_**R**_(×10^−3^)	699.08	485.45	**14.8**
	$\varepsilon_{\vec{t}}\left(\times 10^{-3}\right)$	26.68	25.52	**4.195**

**Fig 2:**
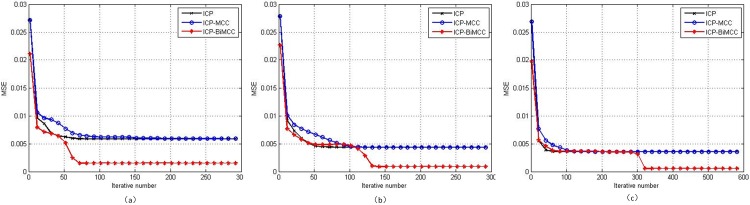
Registration convergences of ICP, ICP-MCC and ICP-BiMCC. (a) ‘Bunny’, (b) ‘Dragon’, (c) ‘Happy Buddha.’

Fig 2 compares the convergence curves of ‘Bunny’, ‘Dragon’ and ‘Happy Buddha’ among three algorithms. It can be seen all three algorithms are converged and the ICP-BiMCC algorithm achieves the smallest registration error.

To clearly and intuitively demonstrate the results, Fig 1 displays the experiments on the point cloud ‘Bunny’. Blue and red points present the model point cloud and the simulated target point cloud, respectively. In the result of Fig 1(b), we can see that the ICP algorithm uses all corresponding points for registration, whose result is inevitably influenced by the noise. The result of Fig 1(c), the ICP-MCC algorithm mismatches at noisy points, since it is difficult to distinguish the noise and outliers in a local minimum region. However, from Fig 1(d) we can see that the registration result of the ICP-BiMCC algorithm is very accurate. And the noise is filtered out clearly.

### 4.2 Testing on partial overlapping data

**Fig 3:**
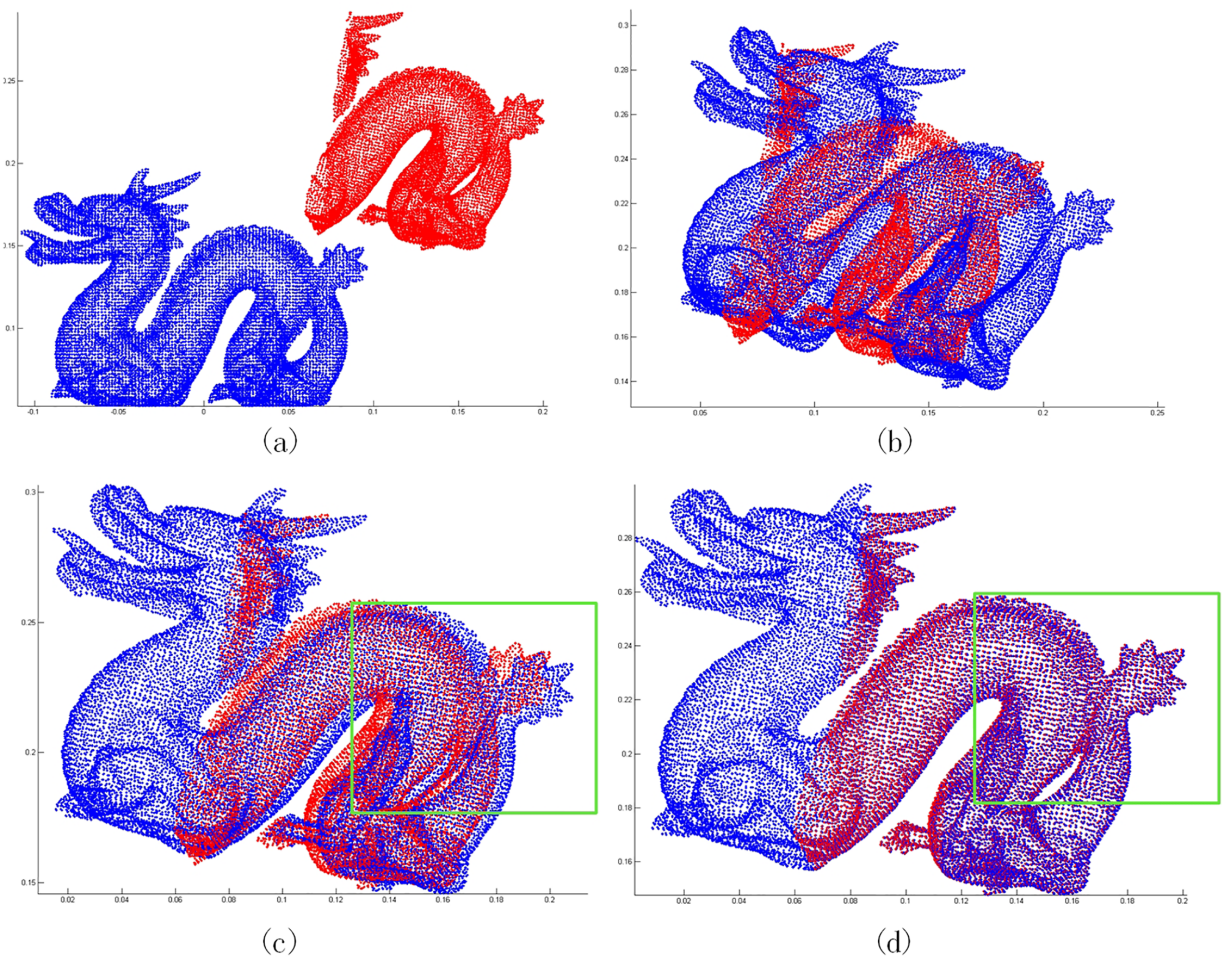
Registration results on noisy ‘dragon’. (a) Initialization. (b) result of ICP. (c) result of ICP-MCC. (d) result of ICP-BiMCC.

**Fig 4:**
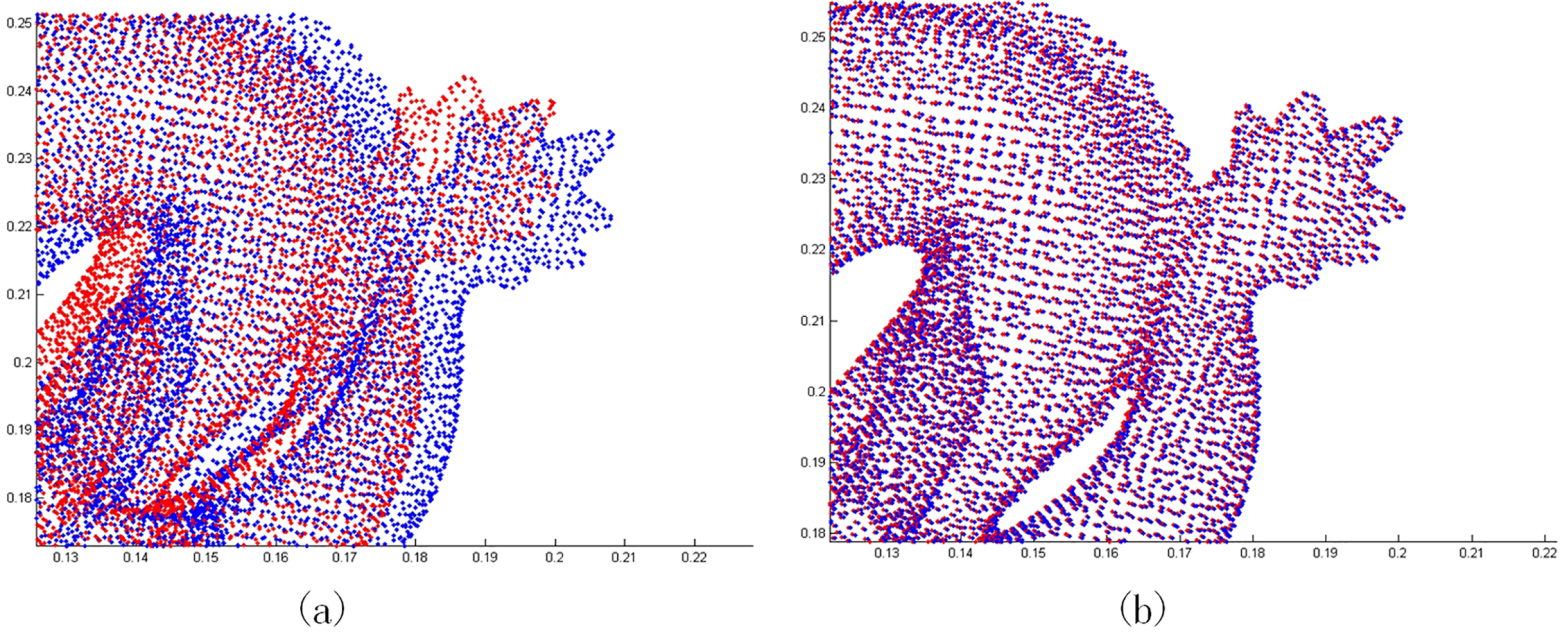
Registration results on the regions marked by green rectangles in Fig 3. (a) result of ICP-MCC. (b) result of ICP-BiMCC.

**Fig 5:**
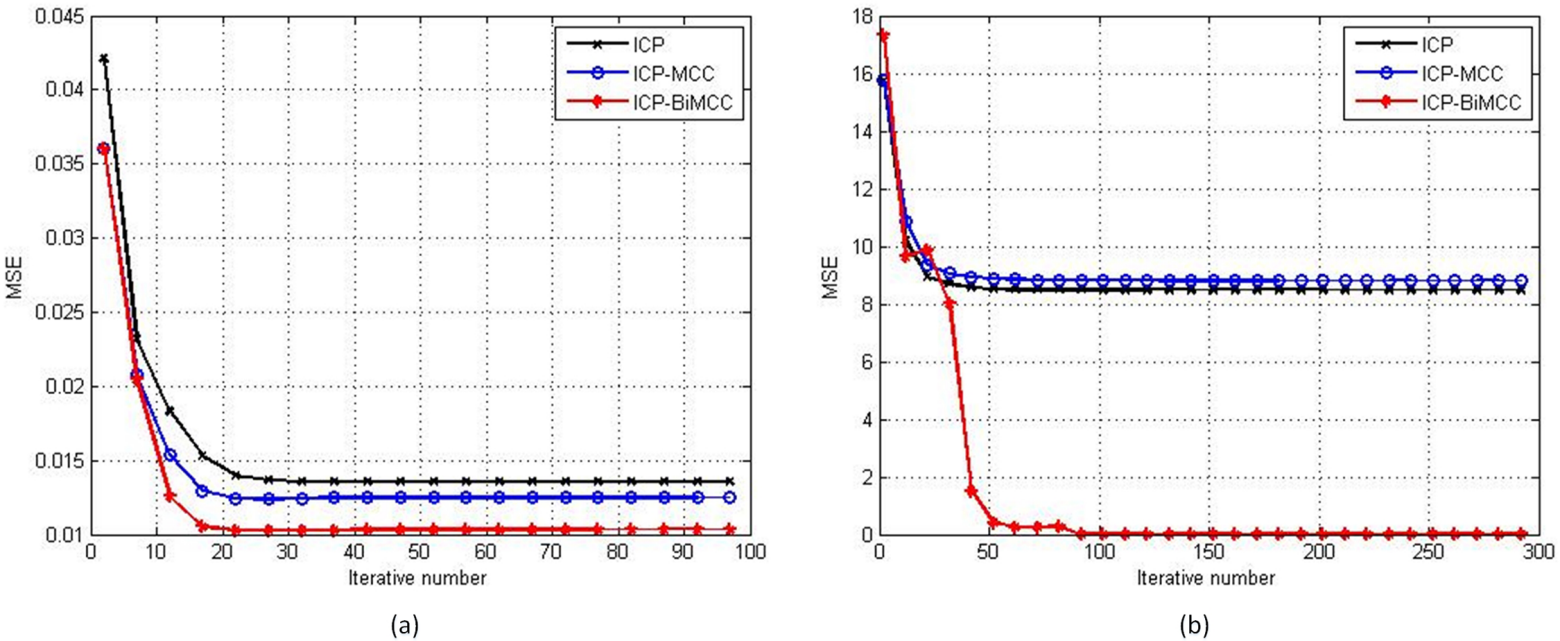
Registration convergence of ICP, ICP-MCC and ICP-BiMCC. (a) ‘Dragon’, (b) ‘T-rex’.

We design two kinds of experiments to test whether the result will be trapped into local minimum on partial overlapping data. In the first experiment, we cut off part of point set. Specifically, the 3D point cloud ‘Dragon’ from S1 Dataset is firstly rotated by 10 degree in each axis and translated by [0.1, 0.1, 0]^*T*^. And then, the upper part of the body, which refers to those points with the first dimension being smaller than -0.03, is trimmed. The differences between the initial point cloud and the target point cloud are shown in Fig 3(a). Fig 3(b)–3(d) show the registration results by three algorithms. From the Fig 3(b), we can find the ICP algorithm can not deal with the registration of partial overlapping accurately. Compared Fig 3(c) and 3(d), we can see that ICP-BiMCC algorithm is more accurate and robust to outliers. To more clearly see the details of the registration region, the regions marked by green rectangles in Fig 3 are amplified in Fig 4. We can clearly see that the registration result of ICP-BiMCC algorithm is more accurate than the ICP-MCC algorithm. Fig 5(a) demonstrates their convergence curves. It can be seen all three algorithms are converged and the ICP-BiMCC algorithm achieves the smallest registration error.

**Fig 6:**
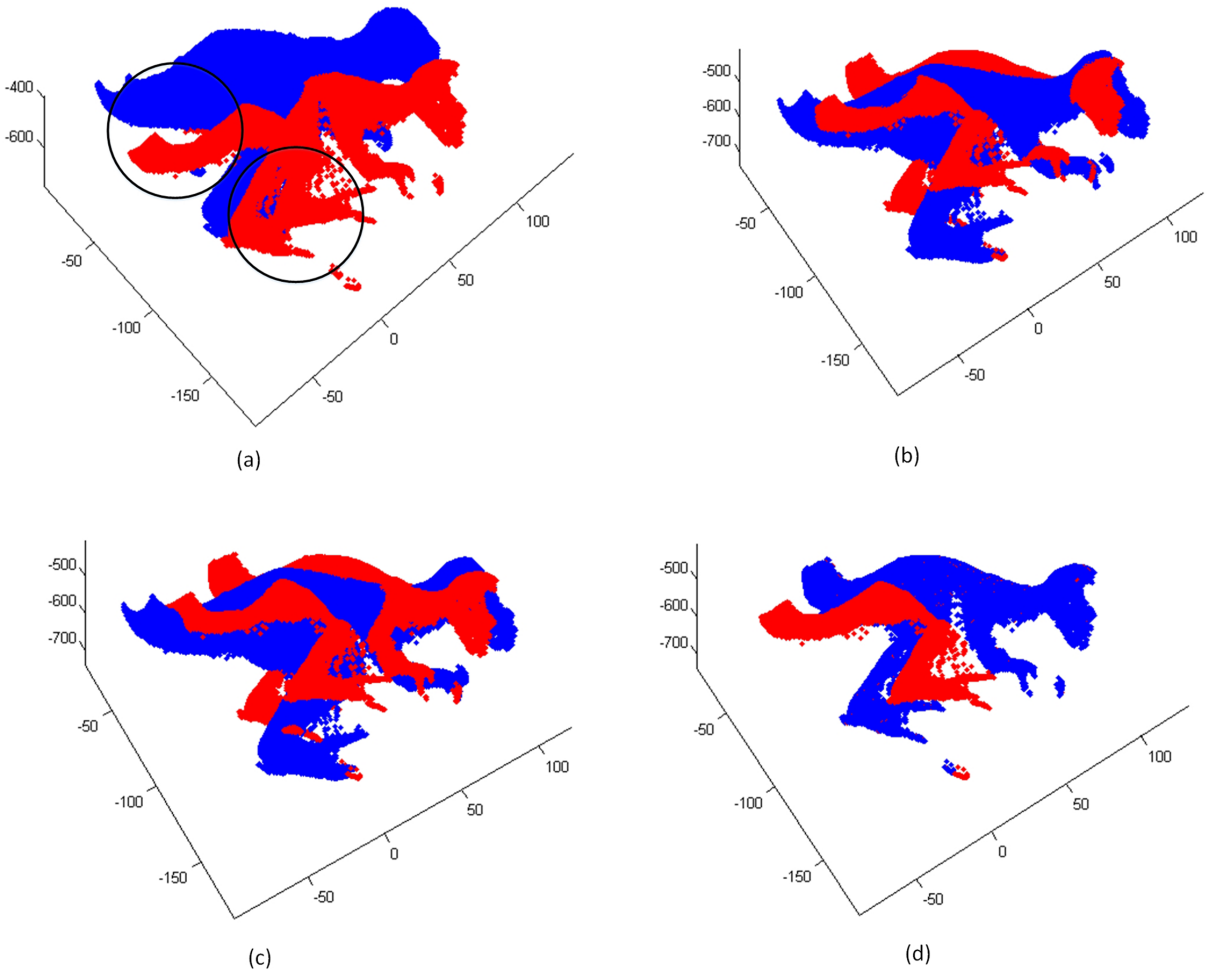
Registration results on noisy ‘T-rex’. (a) Initialization. (b) result of ICP. (c) result of ICP-MCC. (d) result of DICP-MCC.

In the second experiment, we add some local similar shapes to the original data to test the robustness of the algorithm. During the iterative process, since their local similarity, when the result reaches the partial registration, the other part will be easily considered as outliers. But more robust algorithm can avoid it. Specifically, one more leg and one more tail of 3D point cloud ‘T-rex’ from from S1 Dataset in [30] are copied, and given a rotation and a translation transformation, then added to the original point cloud. The differences between the initial point cloud and the simulated data are pointed out by the circles in Fig 6(a). Fig 6(b)–6(d) show the registration results by three algorithms. We can see that the results of ICP and ICP-MCC algorithm are mismatched because of the additional part. However, the ICP-BiMCC algorithm can find the maximum overlapping parts and register two point clouds accurately. Fig 5(b) demonstrate their convergence curves. It can be seen all three algorithms are converged and the ICP-BiMCC algorithm achieves the smallest registration error.

## 5 Conclusions

This paper presents a robust point cloud registration algorithm based on the BiMCC. The proposed formulation replaces the similarity measure to the BiMCC loss, which is more robust to the noise and outliers in practice and avoid the registration result being trapped into a local minimum caused by bad initializations. The fixed-point optimization technique under the ICP framework is adopted to solve the proposed formulation. Qualitative and quantitative experimental results under noisy conditions show that our approach outperforms other related methods.

## Supporting information

S1 Dataset The minimal data set. (ZIP)
